# Prognostic value of receptor conversion after neoadjuvant chemotherapy in breast cancer patients: a prospective observational study

**DOI:** 10.18632/oncotarget.3292

**Published:** 2015-03-16

**Authors:** Xi Jin, Yi-Zhou Jiang, Sheng Chen, Ke-Da Yu, Zhi-Ming Shao, Gen-Hong Di

**Affiliations:** ^1^ Department of Breast Surgery, Fudan University Shanghai Cancer Center, Department of Oncology, Shanghai Medical College, Fudan University, Shanghai 200032, China

**Keywords:** breast cancer, neoadjuvant chemotherapy, receptor conversion, prognosis

## Abstract

The hormone receptor (HR) status and human epidermal growth hormone receptor 2 (HER2) status of patients with breast cancer may change following neoadjuvant chemotherapy (NCT). This prospective observational study aimed to evaluate the prognostic impact of receptor conversion in breast cancer patients treated with NCT.Of the 423 consecutive patients who had residual disease in the breast after NCT, 55 (13.0%) changed from HR (+) to HR (−), 23 (5.4%) changed from HR (−) to HR (+), 27 (6.4%) changed from HER2 (+) to HER2 (−), and 13 (3.1%) changed from HER2 (−) to HER2 (+). A total of 54 (12.8%) changed to the triple-negative (TN) tumor phenotype. The loss of HR positivity was an independent prognostic factor for worse disease-free survival (DFS) and worse overall survival (OS) in multivariate survival analysis. Furthermore, the switch to the TN phenotype after NCT was another independent prognostic factor for worse survival for both DFS and OS. In conclusion, patients with breast cancer may experience changes in HR status, HER2 status and tumor phenotype after NCT. The loss of HR positivity and the switch to the TN phenotype after NCT were associated with a worse patient outcome.

## INTRODUCTION

Neoadjuvant chemotherapy (NCT) followed by definitive surgical resection is a commonly utilized therapeutic approach for locally advanced breast cancer and is likely to improve the operability of these patients by downstaging their primary tumors [1–3]. A core needle biopsy (CNB) is commonly performed to confirm the diagnosis and determine the presence of immunohistochemical (IHC) markers, such as human epidermal growth factor receptor 2 (HER2) and hormone (estrogen and progesterone) receptor (HR), which are key factors in the decision-making process regarding adjuvant therapy as well as important prognostic indicators [4, 5].

Previous studies have shown that NCT can alter the status of HR [6–11] and HER2 [8, 10–13]. Patients showing a conversion from HR (+) to HR (−) tended to benefit less from NCT compared to those with no change or the opposite conversion [from HR (−) to HR (+)] [6]. However, little information is available on the prognostic impact of receptor conversion caused by NCT. The purpose of our study was to assess the discordance rate of the HR status and the HER2 status in patients with residual tumors after NCT and to evaluate the prognostic significance of multiple changes in these statuses.

## RESULTS

### Patient characteristics

A total of 121 of the 544 patients (22.2%) with primary breast cancer who received NCT in our study were considered pathologic complete response (pCR) after NCT. These complete responders were excluded from this prospective observational study due to the lack of residual tumors. The remaining 423 patients (77.8%) with residual disease in the breast were included in this study (Supplemental Figure 1). Of the 423 patients, 351 patients (83.0%) were older than 40 years and 177 patients (41.8%) were post-menopausal. The median tumor size of the surgical specimens was three centimeters, and the average number of lymph nodes involved was four. All patients underwent one to six cycles of NCT using a regimen of PC (Paclitaxel and Carboplatin, 24.8%), CEF (Cyclophosphamide, Epirubicin and 5-fluorouracil, 28.1%), NE (Navelbine and Epirubicin, 39.9%), TE (Docetaxel and Epirubicin, 6.1%), or other agents (0.9%). Approximately 146 patients (53.2%) had high Ki67 expression (Table 1).

**Table 1:** Patients and tumor characteristics

Variables	*n*	%
**Age**		
≤ 40 years	72	17.0
> 40 years	351	83.0
**Menopausal Status**		
pre-menopausal	246	58.2
post-menopausal	177	41.8
**Initial Tumor Size**		
≤ 2 cm	6	1.4
> 2 and ≤ 5 cm	114	27.0
> 5 cm	198	46.8
unknown	105	24.8
**Initial Tumor Stage**		
T1	4	0.9
T2	134	31.7
T3	212	50.1
T4	73	17.2
**Initial Node Status**		
negative	107	25.3
positive	316	74.7
**Histologic Type**		
ductal	386	91.3
lobular	8	1.9
other	29	6.9
**Initial HR Status**		
negative	166	39.2
positive	257	60.8
**Initial HER2 Status**		
negative	341	80.6
positive	82	19.4
**Pre-NCT Tumor Phenotype**		
HR (+)/HER2 (−)	213	50.3
HR (+)/HER2 (+)	44	10.4
HR (−)/HER2 (+)	38	9.0
HR (−)/HER2 (−)	128	30.3
**NCT Regimen**		
PC	105	24.8
CEF	119	28.1
NE	169	39.9
TE	26	6.1
others	4	1.0
**NCT Cycles**		
1–2	106	25.1
3–4	272	64.3
5–6	45	10.6
**Response to NCT**		
PR	229	54.1
SD/PD	194	45.9
**Tumor Size at Surgery**		
≤ 2 cm	152	35.9
> 2 and ≤ 5 cm	206	48.7
> 5 cm	65	15.4
**Number of Positive Nodes at Surgery**		
0	76	18.0
1–3	127	30.0
≥ 4	220	52.0
**Vascular Invasion**		
negative	230	54.4
positive	88	20.8
unknown	105	24.8
**Histologic Grade**		
1	3	0.7
2	159	37.6
3	42	10.0
unknown	219	51.8
**Ki67 at Surgery**		
high (≥ 15%)	146	34.5
low (< 15%)	277	65.5
**HR Status at Surgery**		
negative	198	46.8
positive	225	53.2
**HER2 Status at Surgery**		
negative	355	83.9
positive	68	16.1
**Adjuvant Chemotherapy**		
Yes	377	89.1
No	46	10.9
**Adjuvant Hormone Therapy**		
Yes	230	54.4
No	193	45.6
**Adjuvant Radiotherapy**		
Yes	281	66.4
No	142	33.6
**HR Conversion**		
(−) to (−)	143	33.8
(+) to (+)	202	47.8
(+) to (−)	55	13.0
(−) to (+)	23	5.4
**HER2 Conversion**		
(−) to (−)	328	77.5
(+) to (+)	55	13.0
(+) to (−)	27	6.4
(−) to (+)	13	3.1
**Tumor Phenotype Conversion**		
concordant nTN	213	50.3
concordant TN	110	26.0
discordant nTN	46	10.9
discordant TN	54	12.8

CEF: Cyclophosphamide + Epirubicin + 5-fluorouracil; HER2: human epidermal growth factor receptor 2; HR: hormone receptor; NCT: neoadjuvant chemotherapy; NE: Navelbine + Epirubicin; nTN: non-triple-negative; PC: Paclitaxel + Cyclophosphamide; PR: partial response; SD/PD: stable disease or progression of disease; TE: Docetaxel + Epirubicin; TN: triple-negative.

**Figure 1:**
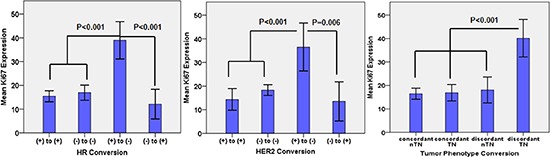
Comparison of Ki-67 expression in the different conversion groups The data represent the mean ± standard deviation of Ki67 levels. Statistical significance is indicated.

We also compared the expression of Ki67 in different receptor conversion groups. Significantly high Ki67 expression was observed in the groups with loss of HR status (mean, 39.0), loss of HER2 status (mean, 36.5) and a discordant triple-negative (TN) tumor phenotype (mean, 30.2) (Figure 1).

### Discordance in receptor expression measurement

Patients with HR and HER2 status conversions before and after NCT were divided into four groups: (+) to (+), (−) to (−), (+) to (−) and (−) to (+). The HR status of up to 78 patients (18.4%) was converted after NCT, and this conversion was predominantly from HR (+) to (−) (55 patients, 13.0%). A total of 23 patients (5.4%) showed a change in their HR status from (−) to (+). With regard to HER2 status, 40 patients (9.5%) presented a discordant HER2 status; of these individuals, 27 (6.3%) were converted from HER2 (+) to HER2 (−), and 13 (3.1%) changed from HER2 (−) to HER2 (+). We defined the tumor phenotypes as HR (+)/HER2 (−), HR (+)/HER2 (+), HR (−)/HER2 (+) and HR (−) /HER2 (−). Discordance in tumor phenotypes was observed in 100 patients (23.6%), and 54 patients (12.8%) converted to TN (Table 1).

### Prognostic impact of HR and HER2 conversion

**Figure 2:**
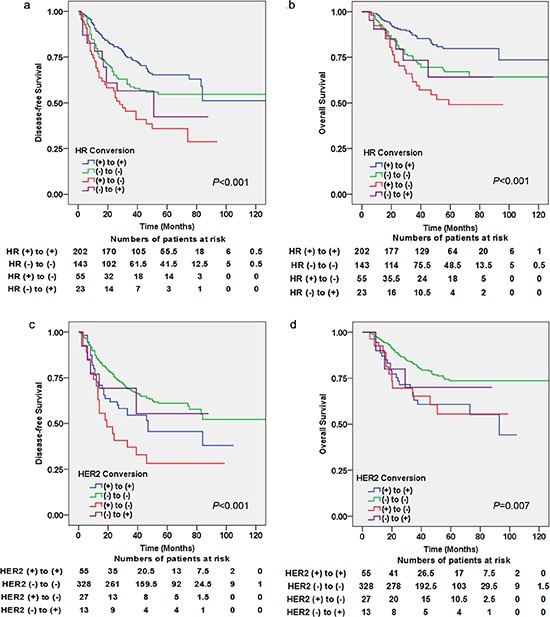
Kaplan–Meier estimates of disease-free survival (DFS) and overall survival (OS) according to receptor conversions **(a)** DFS for hormone receptor (HR) conversion (log-rank test: *P* < 0.001), **(b)** DFS for human epidermal growth factor receptor 2 (HER2) conversion (log-rank test: *P* < 0.001), **(c)** OS for HR conversion (log-rank test: *P* < 0.001), **(d)** OS for HER2 conversion (log-rank test: *P* = 0.007).

Kaplan–Meier plots for disease-free survival (DFS) and overall survival (OS) according to HR conversion and HER2 conversion are shown in Figure 2. Patients showing a change in their HR status from (+) to (−) after NCT had significantly worse DFS than the other three groups of patients (*P* < 0.001) (Figure 2a). Similar significant differences in OS were also observed (*P* < 0.001) (Figure 2b). According to the four HER2 conversion groups, patients who remained HER2 (−) after NCT had better DFS (*P* < 0.001) (Figure 2c) and OS (*P* = 0.007) (Figure 2d).

To further evaluate the difference in patient survival based on changes in their HR and HER2 statuses after NCT, univariate and multivariate Cox regression analyses of DFS and OS were carried out. The results of univariate Cox regression analysis are shown in Supplemental Table 1. The multivariate model included all variables that were statistically significant in the univariate analysis except for interactive variables (Table 2). In the multivariate Cox regression analysis, the number of positive nodes at surgery (*P* < 0.001 and *P* = 0.001), HR conversion (*P* = 0.001 and *P* = 0.001) and HER2 conversion (*P* < 0.001 and *P* = 0.003) were statistically significant for both DFS and OS; NCT regimens were only significant for OS (*P* = 0.002). Among the groups showing a change in HR status, patients with loss of HR positivity had significantly worse survival outcomes (Hazard ratios, HazR = 2.648, *P* < 0.001 for DFS; HazR = 3.460, *P* < 0.001 for OS) compared with positive patients with a concordant HR status (HazR = 1 for DFS and OS) and patients with an HR status gain (HazR = 2.400, *P* = 0.013 for DFS; HazR = 3.834, *P* = 0.008 for OS). Loss of HER2 positivity alone was not significantly associated with DFS or OS.

**Table 2:** Multivariate analyses of DFS and OS of non-pCR patients with neoadjuvant chemotherapy (*n* = 423)

Variables	ALL		Multivariate analyses			
			DFS^a^		OS^b^	
	*n*	%	*P*	HazR (95% CI)	*P*	HazR (95% CI)
**HR Conversion**			0.001		0.001	
(+) to (+)	202	47.8		1		1
(−) to (−)	143	33.8	0.411	1.268(0.720–2.235)	0.596	1.222(0.583–2.558)
(+) to (−)	55	13.0	< 0.001	2.648(1.609–4.358)	< 0.001	3.460(1.738–6.885)
(−) to (+)	23	5.4	0.013	2.400(1.206–4.776)	0.008	3.834(1.416–10.377)
**HER2 Conversion**			< 0.001		0.003	
(+) to (+)	55	13.0		1		1
(−) to (−)	328	77.5	0.033	0.609(0.386–0.961)	0.005	0.434(0.243–0.776)
(+) to (−)	27	6.4	0.058	1.856(0.980–3.515)	0.687	1.184(0.520–2.695)
(−) to (+)	13	3.1	0.299	0.561(0.189–1.671)	0.248	0.399(0.084–1.895)
**Tumor Phenotype Conversion**			< 0.001		0.001	
concordant nTN	213	50.3		1		1
concordant TN	110	26.0	0.545	0.862(0.532–1.395)	0.527	0.825(0.454–1.498)
discordant nTN	46	10.9	0.060	1.691(0.978–2.922)	0.027	2.282(1.099–4.739)
discordant TN	54	12.8	< 0.001	2.713(1.718–4.284)	0.003	2.477(1.373–4.467)

CI: confidence interval; DFS: disease-free survival; HazR: hazard ratio; HER2: human epidermal growth factor receptor 2; HR: hormone receptor; nTN: non-triple-negative; OS: overall survival; TN: triple-negative.

a The DFS multivariate analyses were adjusted for initial node status, NCT cycles, response to NCT, tumor size at surgery, number of positive nodes at surgery and adjuvant hormone therapy.

b The OS multivariate analyses were adjusted for initial tumor status, initial node status, NCT regimens, tumor size at surgery, number of positive nodes at surgery and adjuvant hormone therapy.

To exclude the influence of hormone therapy, we next performed Kaplan–Meier plots to analyze the prognostic impact of HR conversion among 190 patients who received hormone therapy. Patients with loss of HR status after NCT still had significantly worse survival outcomes compared with patients with a concordant HR positive status (*P* = 0.002 for DFS and *P* = 0.003 for OS) (Supplemental Figure 2).

### Tumor phenotype conversion and patient outcomes

**Figure 3:**
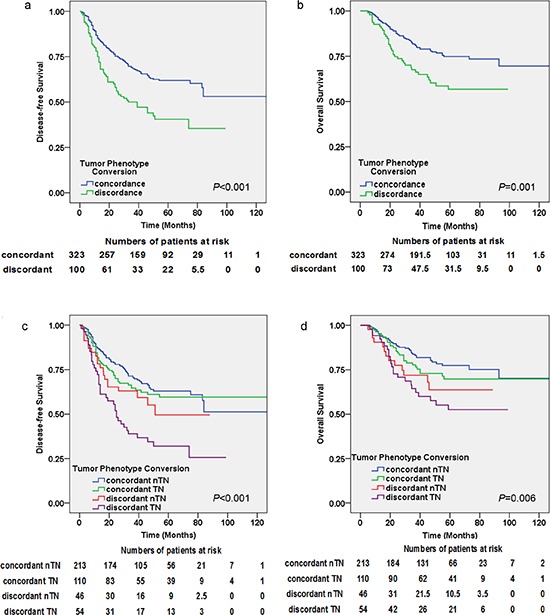
Kaplan–Meier estimates of DFS and OS by tumor phenotype conversion **(a)** DFS (log-rank test: *P* < 0.001), **(b)** OS (log-rank test: *P* = 0.001). Kaplan–Meier estimates of DFS and OS for the groups with concordant triple-negative (TN) group, concordant non-TN (nTN) group, discordant TN group and discordant nTN group: **(c)** DFS (log-rank test: *P* < 0.001), **(d)** OS (log-rank test: *P* = 0.003).

Patients showing a conversion of the tumor phenotype were divided into four groups: concordant non-TN (nTN): the tumor phenotype was unchanged and not TN; concordant TN: the tumor phenotype was unchanged and TN; discordant nTN: the tumor phenotype was changed and the residual tumor was not TN; discordant TN: the tumor phenotype was changed and the residual tumor was TN. We performed Kaplan-Meier analyses of the tumor phenotype conversion (Figure 3). Patients who maintained the same tumor phenotype with no changes had significantly better outcomes compared with discordant cases (*P* < 0.001 for DFS and *P* = 0.001 for OS) (Figure 3a and 3b).

Among these four groups, patients whose tumor phenotype switched to TN had the worst DFS (HazR = 2.713, *P* < 0.001) and OS (HazR = 2.477, *P* = 0.003) compared with patients with concordant nTN (HazR = 1 for DFS and OS) and discordant nTN (HazR = 2.282, *P* = 0.027 for OS, not significant for DFS) in multivariate analyses. Similar results are also seen in Figure 3c and 3d. Patients with concordant TN and concordant nTN did not reach statistical significance for DFS or OS (Table 2).

## DISCUSSION

Previous studies have presented conflicting results regarding the conversion of the HR status and the HER2 status of patients with breast cancer during NCT. While several studies have suggested that the expression of these receptors is altered after NCT [6–13], others indicated that they remained stable [14–17]. Few prospective studies have focused on the prognostic value of a discordant status. In our prospective observational study, we demonstrated that patients showing a conversion from HR (+) to HR (−) in their residual tumors after NCT had a worst outcome (with or without hormonal therapy) compared with other types of HR conversions. The conversion of HER2 status alone did not have a significant impact on the prognosis. A switch to TN breast cancer was associated with a worse outcome compared to that of patients with concordant TN and discordant nTN (only significant for OS).

The mechanism of the conversion of HR and HER2 status after NCT is complex. It is important to note that intratumoral heterogeneity can result in the presence of several different clones with different phenotypes within individual tumors [18, 19]. Within the same tumor, some clones are HR (+), while others are HR (−). Likewise, HER2 (+) cells are also not distributed evenly within individual tumors. The sensitivity to chemotherapy differs between different clones. Tumor cells that are HR (−) are more sensitive to chemotherapy than HR (+) tumors, and HR (+) cells, known as insensitive tumor cells [20–22], are left behind as part of the residual disease after NCT [8, 23]. Likewise, Thor et al [24], Quddus et al [13] and Wang et al [25] have documented that HER2 (+) tumor cells are more likely to be eliminated by chemotherapy, and patients with a high percentage of HER2-positive tumor cells showed a good pathologic response. We concluded that the differential sensitivity to NCT caused by the heterogeneity of clones inside individual tumors may account for the change in HR from (−) to (+) and the change in HER2 from (+) to (−) after NCT.

Another known mechanism of HR status conversion is the downregulation of the estrogenic hormone receptor caused by NCT itself. Bines et al and Rose et al described that chemotherapy can suppress ovarian function and adrenal glands [26, 27], and the decrease in the circulating levels of hormone caused by this suppression may alter the HR status of residual tumors from (+) to (−) after NCT [8]. This mechanism is considered to be the main cause for the switch of HR (+) to HR (−) after NCT. However, false-negative identification of the HR status and the HER2 status in CNB due to intratumoral heterogeneity has been reported previously [28]. The conversion from HR (−) to HR (+) and HER2 (−) to HER2 (+) may be due to the availability of tumor material for CNB because CNB may represent only a small proportion of clones of different phenotypes. Other explanations for the conversion of receptor status include genetic mutations [29, 30], statistical errors and staining techniques [12].

Our research highlighted the prognostic value of the discordance in IHC status and tumor phenotype after NCT using the Kaplan-Meier plots and Cox regression. We assessed the expression of Ki67 in surgical specimens and observed relatively high levels of Ki67 expression in patients with loss of HR status and alteration to the TN phenotype after NCT. Ki67 is known to be a cellular proliferation marker [31, 32], and tumors with high expression of Ki67 exhibit relatively more aggressive behavior [6]. The poor outcome of patients associated with the conversion of HR status and the switch to the TN phenotype after NCT might be the result of a high proportion of proliferating cancer cells and their biological behavior.

Inevitably, our study has several limitations. First, as a prospective observational study, but not a clinical trial, the NCT regimens we used were not uniform. It is not easy to determine whether the receptor conversion is attributable to a special agent or to several ones. Furthermore, our study design did not evaluate the contribution of anti-HER2 therapy. Future studies will be needed to validate the prognostic value of receptor conversion in prospective cohorts. Finally, the molecular mechanisms underlying receptor conversions are uncertain.

In conclusion, our prospective observational study demonstrated the existence of discordance in the HR status and HER2 status after NCT and the negative prognostic impact of the loss of receptor positivity. These findings might help optimize the choice of sequential adjuvant therapy and improve patient survival. The administration of NCT might be the main reason for the change in receptor status, but the mechanism needs to be characterized. In the future, further studies are required to identify the mechanism for this switch in receptor status after NCT and to validate the prognostic impact associated with this switch.

## METHODS

### Case selection

This prospective observational study was initiated in 2003. We enrolled patients who were diagnosed with primary breast cancer and received NCT followed by modified radical mastectomy at Fudan University Shanghai Cancer Center (FUSCC) between January 1, 2003 and December 31, 2009. Patients who had received any type of treatment prior to NCT or who had metastatic disease prior to surgery were not eligible for this study. Cases of bilateral breast cancer, male breast cancer and inflammatory breast cancer were also excluded. For each participant, we collected pre-NCT CNB samples and post-NCT surgical specimens. Out of the 544 patients, we further excluded an additional 121 patients who were considered to have pCR after NCT. In total, 423 eligible patients with residual invasive tumors were included in this prospective observational study.

Data on the medical history, patient characteristics (including age, menopausal status, tumor size, tumor status, node status, histologic type, HR status, HER2 status, Ki67 expression at surgery, tumor phenotype, NCT regimens and cycles, response to NCT, vascular invasion, histologic grade and adjuvant therapies), local and distant extent of disease (evaluated by chest CT, bone scan, abdominal ultrasound, bilateral mammography, breast ultrasound or breast MRI), and pathological assessments of morphological and biological features were collected. CNB was performed to confirm the diagnosis of invasive breast cancer prior to NCT and to evaluate the HR and HER2 status. To minimize the influence of tumor heterogeneity, at least two core samples were obtained in each of the tumors.

All patients were followed up every three months for the first year and every 6 months until death. Follow-up was completed on December 31, 2013. The median length of follow-up was 44 months (range, 2 to 149 months).

This study was approved by the Ethics Committee of FUSCC, and each participant signed an informed consent document.

### Treatment

The patients in our study received an NCT regimen consisting of NE (Navelbine and Epirubicin), CEF (Cyclophosphamide, Epirubicin and 5-fluorouracil), TE (Docetaxel and Epirubicin), PC (Paclitaxel and Carboplatin) or other agents for a median of 3 cycles (range, 1–6 cycles). The pCR was defined as complete disappearance of invasive carcinoma in the breast and regional lymph nodes. The clinical response to NCT was evaluated by physical and imaging examinations according to RECIST 1.1. The clinical response was regarded as a partial response (PR) if the reduction in the greatest tumor diameter exceeded 30%. Tumor reduction of less than 30% or an increase of up to 20% in the greatest diameter was regarded as stable disease (SD). An increase of more than 20% in the greatest diameter of the tumor or the appearance of new disease was regarded as disease progression (PD).

Mastectomy and axillary lymph node dissection were performed within four weeks of the completion of NCT. Additional cycles of chemotherapy, including anthracycline-based and/or taxane-based regimens, were administered after the surgery; a total of six to eight cycles of chemotherapy were completed at the discretion of the treating physician on the basis of clinical and pathologic evaluations after surgery. Radiation therapy was offered at the discretion of the treating radiation oncologist after completion of adjuvant chemotherapy. Five-year standard endocrine therapy (tamoxifen for premenopausal patients, aromatase inhibitor for postmenopausal patients or sequential tamoxifen and aromatase inhibitor) was administered to 190 patients with HR (+) status pre- or post-NCT. Trastuzumab was recommended for HER2 (+) patients in the adjuvant setting but was not included in any pre-operative treatment.

### Pathology

Immunohistochemical analysis was performed in formalin-fixed, paraffin-embedded tissue sections using standard procedures for breast tumor specimens from CNB and surgical resections. HR status and HER2 status were evaluated before and after NCT, but the Ki67 index was only available for surgical specimens. Each specimen was examined independently by two experienced pathologists. Data regarding the expression of HR and HER2 were collected from a database at the pathological center of FUSCC. The cut-off value for ER positivity and PR positivity was set at 1% of tumor cells with positive nuclear staining. HER2 (+) status was defined as 3(+) according to circumferential membrane-bound staining (HercepTest; Dako Cytomation) or amplification confirmed by fluorescence *in situ* hybridization (FISH). Ki67 expression was divided into two groups: Ki67 index ≥ 15% (high expression) and Ki67 index < 15% (low expression) [6]. The following antibodies were used for IHC: ER (M7047, clone 1D5, Dako, Produktionsvej, Glostrup, Denmark), PR (M3569, clone PgR636, Dako), HER2 (A0485, polyclonal rabbit antibody, Dako) and Ki-67 (M7240, clone MIB-1, Dako).

### Statistical analysis

DFS was calculated from the date of surgery to the date of disease relapse (local, regional or distant relapse), the diagnosis of contralateral breast cancer or death from any cause. OS was calculated from the date of diagnosis to the date of death or last follow-up. Survival curves were estimated using the Kaplan–Meier method, and the log-rank test was used to test for differences between groups. HazR and their 95% confidence intervals (CIs) were calculated using the Cox regression model. Multivariate Cox proportional hazard analyses were performed by adjusting for possible prognostic variables (*P* < 0.05 in univariate analysis) using a stepwise selection method. One-way ANOVA was used to evaluate the differences in variables among multiple groups. The Bonferroni test was performed when necessary. The results were considered statistically significant if the *P* was < 0.05. The statistical analysis was carried out using SPSS (version 20.0; SPSS Company, Chicago, IL).

## SUPPLEMENTARY FIGURES AND TABLE


