# Supplementary material for: Prognostic value of receptor conversion after neoadjuvant chemotherapy in breast cancer patients: a prospective observational study
Source: Oncotarget. 2015 Mar 16;6(11):9600–11. doi: 10.18632/oncotarget.3292 (PMC4496242; doi:10.18632/oncotarget.3292)
Supplement: Supplementary file 1 [file oncotarget-06-9600-s001.pdf]

SUPPLEMENTARY FIGURES AND TABLE

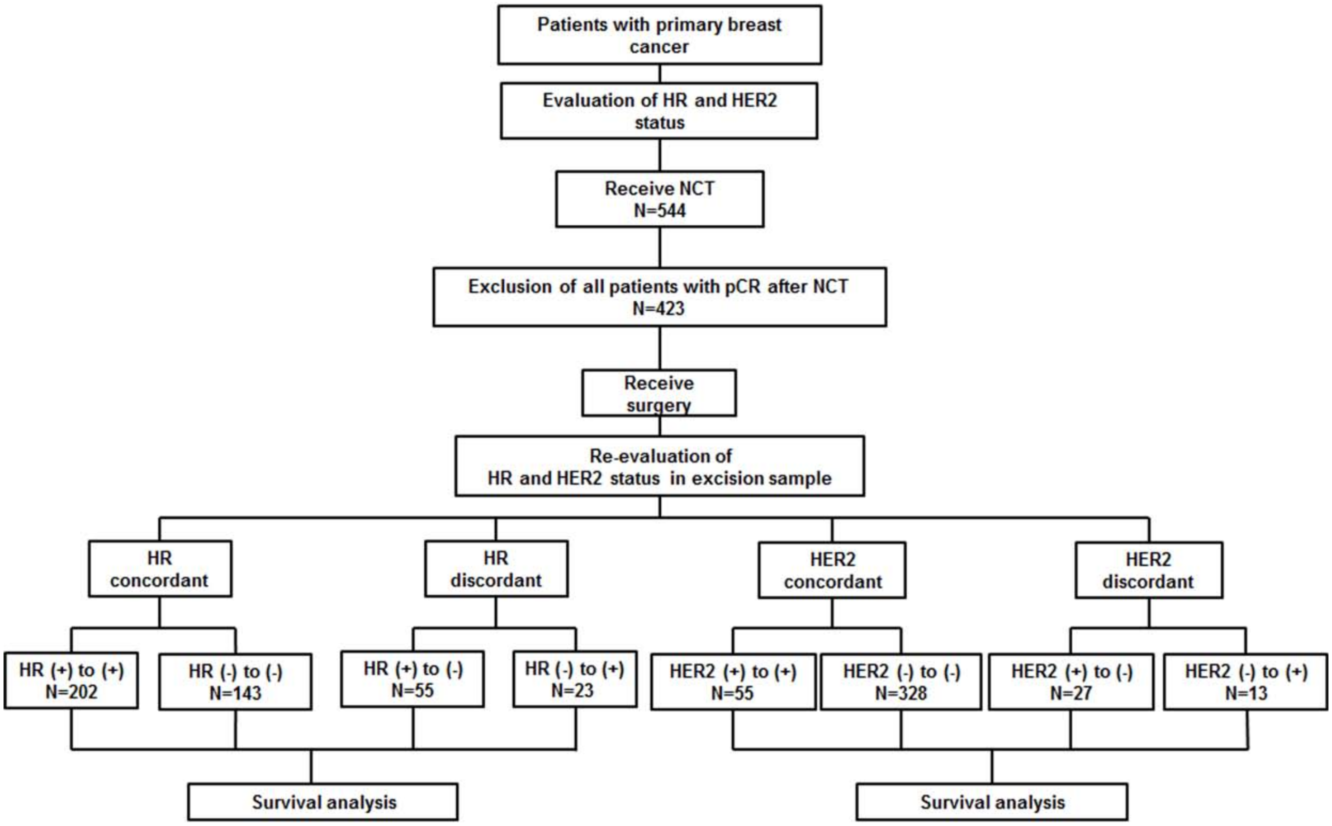

Supplementary Figure S1: Flow diagram of the study design. A total of 423 patients who received neoadjuvant chemotherapy (NCT) and had residual tumors were included in this study.

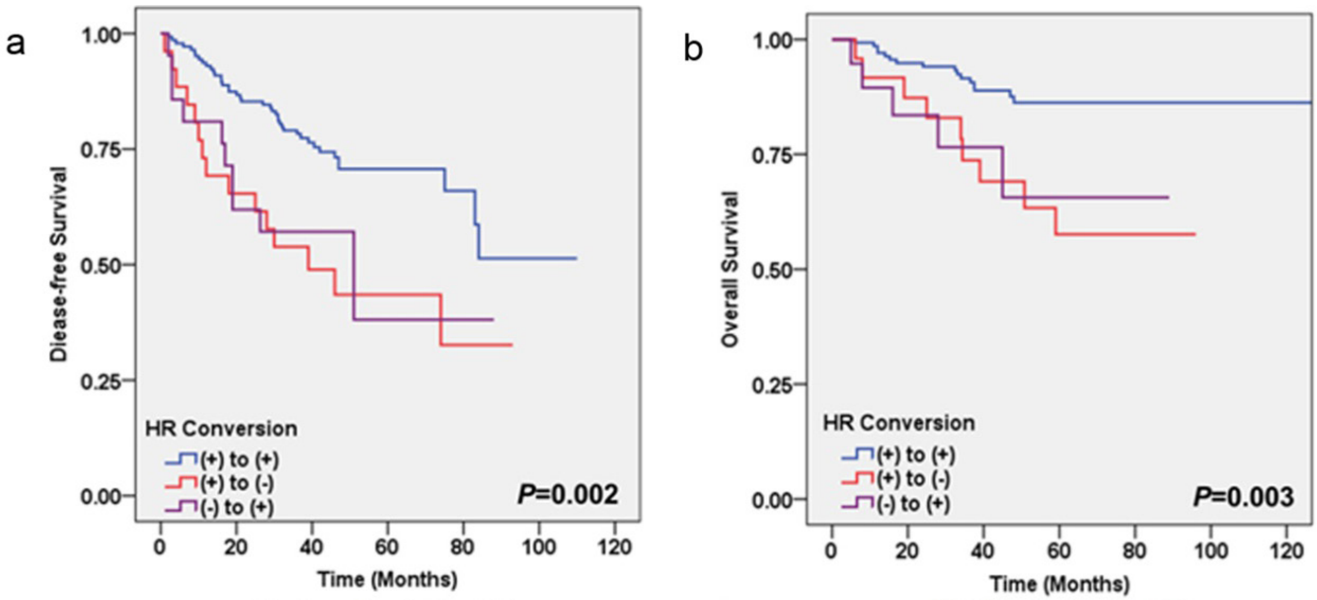

Numbers of patients at risk

HR(+) to (+)	143	125	76	36	10	2	0
HR(+) to (-)	26	17	10	8	2	0	0
HR(-) to (+)	21	13	6	2	1	0	0

Numbers of patients at risk

HR(+) to (+)	143	126	94	44	12	2	1
HR(+) to (-)	26	20	14	10	4	0	0
HR(-) to (+)	21	14	10	3	2	0	0

Supplementary Figure S2: Kaplan-Meier estimates of DFS and OS of HR conversion among 190 hormone therapy-administered patients a. DFS (log-rank test: *P* = 0.002), b. OS (log-rank test: *P* = 0.003).

Supplementary Table S1: Univariate analyses of DFS and OS of non-pCR patients with neoadjuvant chemotherapy ($n = 423$)

Variables	ALL		Univariate analysis			
			DFS		OS	
	n	%	*P*	HazR(95%CI)	*P*	HazR(95%CI)
**Age**			0.946		0.563	
≤ 40 years	72	17.0				
> 40 years	351	83.0				
**Menopausal Status**			0.239		0.289	
Pre	246	58.2				
Post	177	41.8				
**Initial Tumor Status**			0.058		0.031	
T1	4	0.9				1
T2	134	31.7			0.759	0.732(0.099–5.401)
T3	212	50.1			0.898	1.138(0.157–8.220)
T4	73	17.2			0.590	1.734(0.234–12.857)
**Initial Node Status**			0.049		0.018	
negative	107	25.3		1		1
positive	316	74.7	0.049	1.444(1.001–2.083)	0.018	1.867(1.111–3.137)
**Initial HR Status**			0.220		0.091	
negative	166	39.2				
positive	257	60.8				
**Initial HER2 Status**			< 0.001		0.001	
negative	341	80.6		1		1
positive	82	19.4	< 0.001	1.830(1.313–2.551)	0.001	2.028(1.344–3.060)
**NCT Regimens**			0.077		0.035	
PC	105	24.8				1
CEF	119	28.1			0.006	2.472(1.294–4.723)
NE	169	39.9			0.099	1.721(0.903–3.277)
TE	26	6.1			0.125	2.018(0.823–4.952)
others	4	0.9			0.028	5.384(1.204–24.083)
**NCT Cycles**			0.006		0.125	
1–2	106	25.1		1		
3–4	272	64.3	0.121	0.765(0.545–1.073)		
5–6	45	10.6	0.081	1.526(0.949–2.454)		

(*Continued*)

Variables	ALL		Univariate analysis			
			DFS		OS	
	n	%	*P*	HazR(95%CI)	*P*	HazR(95%CI)
**Response to NCT**			0.028		0.090	
PR	229	54.1		1		
SD/PD	194	45.9	0.028	1.392(1.036–1.870)		
**Tumor Size at Surgery**			0.001		0.049	
≤ 2 cm	152	35.9		1		1
> 2 and ≤ 5cm	206	48.7	0.197	1.255(0.889–1.771)	0.660	1.104(0.710–1.718)
> 5cm	65	15.4	< 0.001	2.155(1.422–3.265)	0.021	1.859(1.099–3.147)
**Number of Involved Nodes at Surgery**			< 0.001		< 0.001	
0	76	18.0		1		1
1–3	127	30.0	0.155	1.496(0.859–2.604)	0.175	1.706(0.789–3.688)
≥ 4	220	52.0	< 0.001	2.849(1.734–4.682)	0.001	3.327(1.665–6.650)
**Vascular Invasion**			0.087		0.081	
negative	230	54.4				
positive	88	20.8				
unknown	105	24.8				
**Histologic Grade**			0.670		0.519	
1	3	0.7				
2	159	37.6				
3	42	10.0				
unknown	219	51.8				
**Ki67 at Surgery**			0.465		0.856	
High (≥ 15%)	146	34.5				
Low (< 15%)	229	54.1				
unknown	48	11.3				
**HR Status at Surgery**			0.001		< 0.001	
negative	198	46.8		1		1
positive	225	53.2	0.001	0.617(0.459–0.830)	< 0.001	0.491(0.331–0.728)
**HER2 Status at Surgery**			0.109		0.009	
negative	355	83.9				1
positive	68	16.1			0.009	1.187(1.160–2.847)

(*Continued*)

Variables	ALL		Univariate analysis			
			DFS		OS	
	n	%	*P*	HazR(95%CI)	*P*	HazR(95%CI)
**Adjuvant Chemotherapy**			0.064		0.724	
No	62	14.7				
Yes	313	73.9				
unknown	48	11.3				
**Adjuvant Hormone Therapy**			0.025		0.001	
No	185	43.7		1		1
Yes	190	44.9	0.025	0.691(0.501–0.955)	0.001	0.477(0.306–0.742)
unknown	48	11.3				
**Adjuvant Radiotherapy**			0.464		0.986	
No	139	32.9				
Yes	236	55.8				
unknown	48	11.3				
**HR Conversion**			< 0.001		< 0.001	
(+) to (+)	202	47.8		1		1
(−) to (−)	143	33.8	0.026	1.475(1.047–2.079)	0.005	1.904(1.211–2.993)
(+) to (−)	55	13.0	< 0.001	2.497(1.660–3.757)	< 0.001	3.087(1.830–5.208)
(−) to (+)	23	5.4	0.075	1.785(0.944–3.377)	0.089	2.123(0.891–5.060)
**HER2 Conversion**			0.001		0.009	
(+) to (+)	55	13.0		1		1
(−) to (−)	328	77.5	0.032	0.641(0.428–0.961)	0.003	0.488(0.302–0.788)
(+) to (−)	27	6.4	0.102	1.621(0.908–2.893)	0.984	1.007(0.488–2.079)
(−) to (+)	13	3.1	0.513	0.729(0.282–1.883)	0.581	0.712(0.213–2.379)
**Tumor Phenotype Conversion**			< 0.001		0.007	
concordant nTN	213	50.3		1		1
concordant TN	110	26.0	0.508	1.134(0.781–1.648)	0.224	1.346(0.834–2.171)
discordant nTN	46	10.9	0.177	1.404(0.858–2.298)	0.055	1.838(0.986–3.426)
discordant TN	54	12.8	< 0.001	2.471(1.668–3.660)	0.001	2.356(1.416–3.918)

CEF: Cyclophosphamide + Epirubicin + 5-fluorouracil; CI: confidence interval; DFS: disease-free survival; HazR: hazard ratio; HER2: human epidermal receptor 2; HR: hormone receptor; NCT: neoadjuvant chemotherapy; NE: Navelbine + Epirubicin; nTN: non-triple negative; OS: overall survival; PC: Paclitaxel + Carboplatin; PR: partial response; SD/PD: stable disease or progression of disease; TE: Docetaxel + Epirubicin; TN: triple-negative.
